# Laboratory-acquired infections of *Salmonella enterica* serotype Typhi in South Africa: phenotypic and genotypic analysis of isolates

**DOI:** 10.1186/s12879-017-2757-2

**Published:** 2017-09-29

**Authors:** Anthony Marius Smith, Shannon Lucrecia Smouse, Nomsa Pauline Tau, Colleen Bamford, Vineshree Mischka Moodley, Charlene Jacobs, Kerrigan Mary McCarthy, Adré Lourens, Karen Helena Keddy, GERMS-SA Surveillance Network, Vanessa Quan, Ananta Nanoo, Anne von Gottberg, Andries Dreyer, Anthony Smith, Arvinda Sooka, Cecilia Miller, Charlotte Sriruttan, Cheryl Cohen, Chikwe Ihekweazu, Claire von Mollendorf, Frans Radebe, Genevie Ntshoe, Gillian Hunt, Karen Keddy, Linda de Gouveia, Linda Erasmus, Marshagne Smith, Martha Bodiba, Mbhekiseni Khumalo, Motshabi Modise, Nazir Ismail, Nelesh Govender, Nicola Page, Olga Perovic, Oliver Murangandi, Penny Crowther-Gibson, Portia Mutevedzi, Riyadh Manesen, Ruth Mpembe, Samantha Iyaloo, Sarona Lengana, Shabir Madhi, Sibongile Walaza, Sonwabo Lindani, Susan Meiring, Thejane Motladiile, Verushka Chetty Carel Haumann, Patricia Hanise, Sandeep Vasaikar, John Black, Vanessa Pearce, Anwar Hoosen, Vicky Kleinhans, Alan Karstaedt, Caroline Maluleka, Charl Verwey, Charles Feldman, David Moore, David Spencer, Gary Reubenson, Khine Swe Swe Han, Jeannette Wadula, Jeremy Nel, Kathy Lindeque, Maphoshane Nchabeleng, Nicolette du Plessis, Norma Bosman, Ranmini Kularatne, Ruth Lekalakala, Sharona Seetharam, Theunis Avenant, Trusha Nana, Vindana Chibabhai, Adhil Maharj, Asmeeta Burra, Fathima Naby, Halima Dawood, Koleka Mlisana, Lisha Sookan, Praksha Ramjathan, Prasha Mahabeer, Romola Naidoo, Sumayya Haffejee, Yacoob Coovadia, Ken Hamese, Ngoaka Sibiya, Greta Hoyland, Jacob Lebudi, Eunice Weenink, Riezaah Abrahams, Sindiswa Makate, Ebrahim Variava, Erna du Plessis, Andrew Whitelaw, Catherine Samuel, Mark Nicol, Preneshni Naicker, Shareef Abrahams, Adrian Brink, Elizabeth Prentice, Inge Zietsman, Maria Botha, Peter Smith, Xoliswa Poswa, Chetna Govind, Keshree Pillay, Suzy Budavari, Catherine Samuel, Marthinus Senekal, Cynthia Whitney, Keith Klugman

**Affiliations:** 1 Centre for Enteric Diseases, National Institute for Communicable Diseases, National Health Laboratory Service, Private Bag X4, Sandringham, Johannesburg, Gauteng 2131 South Africa; 2 Faculty of Health Sciences, University of the Witwatersrand, Johannesburg, South Africa; 3 National Health Laboratory Service (Groote Schuur Hospital), Cape Town, South Africa; 4 Division of Medical Microbiology, University of Cape Town, Cape Town, South Africa; 5 AMPATH Laboratories, Cape Town, South Africa; 6 Department of Health, Communicable Disease Control, Cape Town, South Africa; 7 Division of Public Health Surveillance and Response, National Institute for Communicable Diseases, National Health Laboratory Service, Johannesburg, South Africa; 8 National Health Laboratory Service (Tygerberg Hospital), Cape Town, South Africa

**Keywords:** *Salmonella* Typhi, Laboratory-acquired infection, South Africa, Genotyping, Molecular subtyping, PFGE, MLST, Whole-genome sequencing, WGS

## Abstract

**Background:**

Workers in clinical microbiology laboratories are exposed to a variety of pathogenic microorganisms. *Salmonella* species is among the most commonly reported bacterial causes of laboratory-acquired infections. We report on three cases of laboratory-acquired *Salmonella enterica* serotype Typhi (*Salmonella* Typhi) infection which occurred over the period 2012 to 2016 in South Africa.

**Methods:**

Laboratory investigation included phenotypic and genotypic characterization of isolates. Phenotypic analysis included standard microbiological identification techniques, serotyping and antimicrobial susceptibility testing. Genotypic analysis included the molecular subtyping methodologies of pulsed-field gel electrophoresis analysis, multilocus sequence typing and whole-genome sequencing (WGS); with WGS data analysis including phylogenetic analysis based upon comparison of single nucleotide polymorphism profiles of isolates.

**Results:**

All cases of laboratory-acquired infection were most likely the result of lapses in good laboratory practice and laboratory safety. The following critical issues were highlighted. There was misdiagnosis and misreporting of *Salmonella* Typhi as nontyphoidal *Salmonella* by a diagnostic laboratory, with associated public health implications. We highlight issues concerning the importance of accurate fluoroquinolone susceptibility testing and interpretation of results according to updated guidelines. We describe potential shortcomings of a single disk susceptibility screening test for fluoroquinolone susceptibility and suggest that confirmatory minimum inhibitory concentration testing should always be performed in cases of invasive *Salmonella* infections. These antimicrobial susceptibility testing issues resulted in inappropriate ciprofloxacin therapy which may have been responsible for failure in clearance of pathogen from patients. *Salmonella* Typhi capsular polysaccharide vaccine was not protective in one case, possibly secondarily to a faulty vaccine.

**Conclusions:**

Molecular subtyping of isolates proved effective to investigate the genetic relatedness of isolates. Molecular subtyping data interpreted together with epidemiological data allowed us to pinpoint the most likely sources for our cases of laboratory-acquired infection.

## Background

Workers in clinical microbiology laboratories are exposed to a variety of pathogenic microorganisms [1, 2]. A review of published literature in 2009, reported that bacteria account for the greatest number of reports of laboratory-acquired infections; *Shigella* species, *Brucella* species, *Salmonella* species, *Mycobacterium tuberculosis* and *Neisseria meningitidis* were the most commonly reported bacterial causes of laboratory-acquired infections [1].

There have been very few reports of laboratory-acquired infections involving *Salmonella enterica* serotype Typhi (*Salmonella* Typhi), however many may go unreported. A PubMed literature search on 1 February 2017, using the key words ‘*Salmonella* Typhi laboratory acquired infection’ found 12 publications (English language) reporting laboratory-acquired *Salmonella* Typhi infections [3–14]. These 12 papers were published over the period 1961 to 1997, so over the last 10 years, to the best of our knowledge, there are no recent publications of laboratory-acquired *Salmonella* Typhi infections. However, more recently, there have been reports of nontyphoidal *Salmonella* (NTS) laboratory-acquired infections. In 2016, a *Salmonella enterica* serotype Typhimurium (*Salmonella* Typhimurium) laboratory-acquired infection was reported from Canada by Alexander and coworkers [15]; while in 2014 and 2012, the Centers for Disease Control and Prevention (CDC) reported on *Salmonella* Typhimurium outbreaks in the USA of which the source of the outbreaks were traced to university teaching microbiology laboratories [16, 17] In 2015, Barker and coworkers reported on a *Salmonella* Enteritidis laboratory-acquired infection [18].

In our present study, we report on three cases of laboratory-acquired *Salmonella* Typhi infection which occurred over the period 2012 to 2016 in South Africa. We demonstrate the usefulness of various molecular subtyping techniques to investigate the source of the infections.

## Methods

### Case reports

Three cases of laboratory-acquired *Salmonella* Typhi infection occurred over the period 2012 to 2016 in South Africa. We describe these as follows.

### Case one

In September 2012, a laboratory technologist working at Laboratory-A presented to her clinician with signs of severe malaise, fever and mild diarrhoea. The technologist had six years of working experience in a clinical microbiology laboratory. Blood cultures were collected for laboratory testing. The diagnostic laboratory identified *Salmonella* using the MALDI Biotyper and the isolate failed to agglutinate in *Salmonella* Typhi-specific antisera. Subsequently, the isolate was reported to the clinician as NTS, susceptible to fluoroquinolones. The patient was treated with seven days of oral ciprofloxacin. The isolate was referred to the Centre for Enteric Diseases (CED) at the National Institute for Communicable Diseases (NICD), for confirmation of identification. The CED confirmed *Salmonella* Typhi with intermediate resistance to fluoroquinolones [ciprofloxacin minimum inhibitory concentration (MIC), 0.25 μg/ml] and susceptibility to azithromycin (MIC, 4 μg/ml). The patient, though recovered, was still excreting *Salmonella* Typhi in her stool and was then treated with a combination of azithromycin and fluoroquinolone for 14 days. Subsequent stools tested negative for *Salmonella* Typhi. The patient, being a laboratory technologist with frequent exposure to *Salmonella* Typhi, had received *Salmonella* Typhi vaccination nine months earlier as part of an occupational health program. On investigation, it was found that this vaccine was part of a batch recalled by the manufacturer, because of potentially low (below specification) antigen content [19] .

### Case two

In February 2016, a trainee clinical pathology resident working on the stool bench at a clinical microbiology laboratory (Laboratory-B), presented to a physician with fever, nausea and vomiting. The patient was admitted to hospital and discharged three days later. *Salmonella* Typhi was cultured from microbiological cultures of blood taken on admission. The isolate was referred to the CED for confirmation of identification. The CED confirmed *Salmonella* Typhi with susceptibility to fluoroquinolones (ciprofloxacin MIC, 0.008 μg/ml) and to azithromycin (MIC, 8 μg/ml). The patient responded well to levofloxacin therapy. Further investigation revealed that the patient had recently serotyped a clinical isolate of *Salmonella* Typhi in the laboratory and had not worn gloves at the time. The patient had one year of working experience in a clinical microbiology laboratory and had not been vaccinated against *Salmonella* Typhi.

### Case three

In October 2016, a laboratory technologist working in a clinical microbiology laboratory (Laboratory-C) started complaining of headaches at work. His colleagues offered him an over the counter analgesic but his symptoms persisted. He reported to a hospital emergency room with symptoms of diarrhoea, worsening headaches, temperatures of 39 °C and severe malaise; he was admitted into hospital. Blood chemistry revealed raised liver enzymes and a C-reactive protein (CRP) reading of 90 mg/L. Blood cultures were collected for laboratory testing; *Salmonella* Typhi was cultured. The diagnostic laboratory determined that the isolate was susceptible to ceftriaxone and the fluoroquinolones (Kirby-Bauer disk susceptibility testing method - pefloxacin disk (5-μg) screening zone size of 25 mm). The patient was commenced on intravenous ciprofloxacin by the treating physician. He received seven days of intravenous ciprofloxacin followed up by a seven-day course of oral ciprofloxacin on discharge. The isolate was referred to the CED for confirmation of identification. The CED confirmed the *Salmonella* Typhi identification and determined that the isolate was intermediately-resistant to fluoroquinolones (ciprofloxacin MIC, 0.25 μg/ml) and susceptible to azithromycin (MIC, 4 μg/ml). *Salmonella* Typhi with the same MICs was isolated from subsequent stool samples, collected 16 days and 19 days after the blood culture sample tested positive. The patient was contacted by the treating private physician and readmitted to hospital where he received five days of intravenous azithromycin. On discharge he was prescribed combination therapy comprising of oral ciprofloxacin and azithromycin for one month. Subsequent stools tested negative for *Salmonella* Typhi. The technologist had 28 months working experience in a clinical microbiology laboratory and had no history of vaccination against typhoid fever. Epidemiological investigation determined that the technologist had been exposed to *Salmonella* Typhi while processing a culture of *Salmonella* Typhi.

### Identification of typhoid fever cases and public health response

Typhoid fever is a notifiable disease in South Africa. Based on laboratory diagnosis, typhoid fever is relatively uncommon in South Africa. Laboratory networks report the isolation of *Salmonella* Typhi to the Outbreak Response Unit of the NICD, who in turn notify the district and provincial communicable diseases coordinators. Cases are investigated through a home visit, interviewed and a case investigation form is completed. Cases are later followed up and include further testing of stool samples. For persons who have had contact with cases, their symptoms are assessed and stool samples are also collected for testing. Details of all cases, including patient occupation are reported to the NICD and recorded in a database. Databases are reviewed to identify cases whose occupation includes laboratory work; data collection includes demographic details, clinical and treatment history and outcome data. For the currently described three cases: all patients came from relatively affluent and hygienic home circumstances, and there was no suggestion of family members being ill or other likely sources of infection; laboratory infection control procedures and laboratory safety policies were reviewed and refresher staff training was conducted; we contacted patients and obtained written informed consent to describe their case.

### Referral of bacterial isolates to the CED

The CED is the national reference centre in South Africa for human infections due to enteric pathogens including: *Salmonella* species*, Shigella* species, *Campylobacter* species, diarrhoeagenic *Escherichia coli*, *Vibrio cholerae* and *Listeria monocytogenes.* Isolates from across South Africa are voluntarily submitted to the CED through national laboratory-based surveillance from >200 clinical microbiology laboratories across the country, in relation to potential outbreaks. The CED proceeds with phenotypic and genotypic characterization of isolates. If required, molecular subtyping of isolates is performed. To determine if these cases of typhoid fever in laboratory-workers were acquired from the isolates to which they were exposed in the laboratory, we identified isolates of *Salmonella* Typhi that were submitted to the CED from the laboratory in which the patient worked during the two months prior to onset of illness and subjected these isolates and the patient’s isolate to phenotypic and genotypic characterization, as described below.

### Phenotypic characterization of bacteria

Bacteria were received on Dorset-Egg transport media [Diagnostic Media Products (DMP), National Health Laboratory Service, Johannesburg, South Africa] and sub-cultured onto 5% Blood Agar (DMP), to check for viability and purity. Cultures were identified using standard phenotypic microbiological identification and serotyping techniques, briefly described as follows. As required, bacterial colonies were identified using the VITEK-2 COMPACT 15 automated microbial identification system (bioMérieux, Marcy-l’Étoile, France). Serotyping was performed according to the White-Kauffmann-Le Minor Scheme. Antimicrobial susceptibility testing was performed using the VITEK-2 COMPACT 15 system (bioMérieux) and the Etest method (bioMérieux). Interpretation of antimicrobial susceptibility data was done in accordance with the Clinical and Laboratory Standards Institute (CLSI) [20].

### PCR for the H58 haplotype of *Salmonella* Typhi

PCR to determine whether *Salmonella* Typhi isolates belonged to the H58 haplotype were performed according to the methodology described by Murgia and coworkers [21].

### Pulsed-field gel electrophoresis (PFGE) analysis of bacteria

PFGE analysis of *Xba*I digested genomic DNA was performed using a Bio-Rad CHEF-DR III electrophoresis system (Bio-Rad Laboratories, Hercules, USA), following a PulseNet protocol [22]. PFGE patterns were analyzed using BioNumerics (version 6.5) Software (Applied Maths, Sint-Martens-Latem, Belgium) with dendrograms of the patterns created using the unweighted pair group method with arithmetic averages, with analysis of banding patterns incorporating the Dice-coefficient at an optimization setting of 1.5% and a position tolerance setting of 1.5%.

### Whole-genome sequencing (WGS) analysis of bacteria

Genomic DNA was isolated from bacteria using the Qiagen QIAamp DNA Mini Kit (Qiagen, Hilden, Germany). DNA libraries were prepared using a Nextera XT DNA Library Preparation Kit (Illumina, San Diego, CA, USA), followed by a 2 × 300 paired-end sequencing runs with 100× coverage using Illumina MiSeq equipment. Raw data generated on the MiSeq was further analyzed using tools available in the CLC Genomics Workbench Software, version 8.5 (Qiagen). Using the ‘Trim Sequences Tool’, sequence reads were trimmed to include quality trimming and ambiguity trimming, and length trimming to discard reads below a length of 50 bases. Trimmed reads were assembled using the ‘De novo Assembly Tool’; the assembly algorithm works by using de Bruijn graphs to produce contiguous (contig) sequences (minimum contig length was set at 200 bases).

### Multilocus sequence typing (MLST) of bacteria

Assembled genome data was analyzed using the ‘multilocus sequence typing (MLST)’ on-line analysis pipeline available at the Center for Genomic Epidemiology (CGE) of the Technical University of Denmark [23]. MLST produces sequence types (STs) based on sequence analysis of seven housekeeping genes (*aroC*, *dnaN*, *hemD*, *hisD*, *purE*, *sucA* and *thrA*), as described at the *Salmonella* MLST database [24].

### Single nucleotide polymorphism (SNP) profiles and phylogenetic analysis of bacteria

Assembled genome data was analyzed using the ‘CSIPhylogeny 1.4’ on-line analysis pipeline available at the CGE [25]. The CSIPhylogeny pipeline uses various publicly available programs and the analysis steps are briefly described as follows: assembled genome data is aligned against a reference genome and single nucleotide polymorphisms (SNPs) are called; SNPs are filtered and qualified; final qualified SNPs for each genome is concatenated to an alignment; phylogeny is then inferred based on a comparison of SNP alignments of strains. SNPs were called by alignment and referencing against a South African strain isolated in 2016 (reference number TCD981492). SNP alignments were analyzed with iTOL software [26] to generate phylogenetic maximum-likelihood trees.

## Results

The results for molecular subtyping of isolates are as follows.

**Fig. 1:**
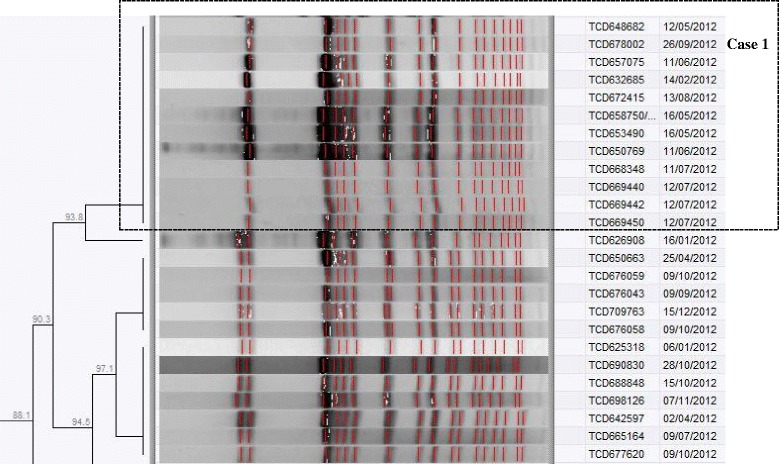
Snapshot from a dendrogram of PFGE (*Xba*I digestion) patterns for all South African isolates of *Salmonella* Typhi (the blocked section indicates a cluster of isolates)

For case one, molecular subtyping using PFGE analysis showed that the PFGE pattern of the patient’s isolate was indistinguishable (100% identical) to that of a PFGE pattern shown by a cluster of *Salmonella* Typhi isolates that the patient (laboratory technologist) had been working on in Laboratory-A (Fig. 1), suggesting that the source of the patient’s infection was within this cluster of isolates that the patient had been working on. This ‘cluster of isolates’ was sourced from a region of South Africa located ~1500 km from where the laboratory technologist lived and worked; also the laboratory technologist had no travel history or any history of contact with persons that lived/travelled to this region of South Africa. Therefore, this could not have been a case of community exposure to *Salmonella* Typhi. For case one, MLST presented a ST 1 subtype for the patient’s isolate; the isolate was also PCR-positive for a marker associated with haplotype H58, a haplotype of *Salmonella* Typhi which is being reported with increasing frequency from many countries in Africa and Asia [27, 28].

**Fig. 2:**
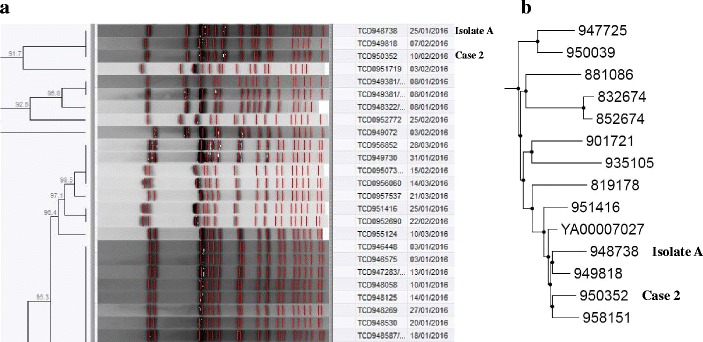
Molecular subtyping of *Salmonella* Typhi isolates. Snapshots are shown from dendrograms and phylogenetic trees of all South African isolates. **a** Snapshot from a dendrogram of PFGE (*Xba*I digestion) patterns (**b**) Snapshot from a phylogenetic maximum-likelihood tree drawn using SNP alignments. Note: isolate 958151 was isolated from a specimen collected on 4 June 2016 (4 months after isolation of the case 2 isolate), so case 2 could not have been exposed to isolate 958151

For case two, molecular subtyping of isolates included PFGE analysis, MLST and SNP profiling. We investigated all *Salmonella* Typhi isolates that the patient (resident) could have been exposed to in Laboratory-B. Compared to three possible isolates, PFGE analysis showed a PFGE pattern match (Fig. 2) to a single isolate (isolate A) from Laboratory-B. Phylogenetic analysis and SNP profiling showed that isolate A was related to case two; the isolates only differed by 41 SNPs (Fig. 2). Both isolates also presented the MLST ST 2 subtype and both were PCR-negative for the marker associated with haplotype H58. These data suggested that the source of the patient’s infection was isolate A. The patient later confirmed that she had indeed worked with isolate A.

**Fig. 3:**
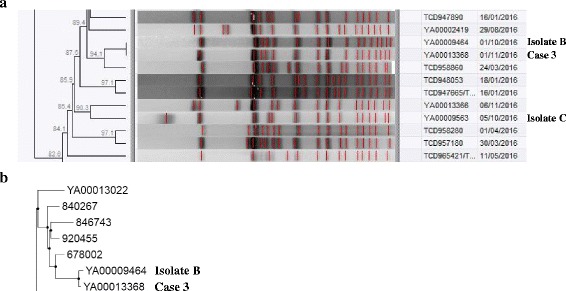
Molecular subtyping of *Salmonella* Typhi isolates. Snapshots are shown from dendrograms and phylogenetic trees of all South African isolates. **a** Snapshot from a dendrogram of PFGE (*Xba*I digestion) patterns (**b**) Snapshot from a phylogenetic maximum-likelihood tree drawn using SNP alignments

For case three, molecular subtyping of isolates included PFGE analysis, MLST and SNP profiling. We investigated all *Salmonella* Typhi isolates that the patient (laboratory technologist) could have been exposed to in Laboratory-C. Compared to two possible isolates (isolates B and C) that the patient could have been exposed to in Laboratory-C; PFGE analysis showed a PFGE pattern match (Fig. 3) to one of the isolates (isolate B). This was supported by MLST; case three presented ST 1 which matched the ST 1 of isolate B; isolate C presented ST 2 and so did not match the case. The case isolate and isolate B were both PCR-positive for the marker associated with haplotype H58. Phylogenetic analysis and SNP profiling confirmed that isolate B was highly related to case three with the isolates only differing by 6 SNPs (Fig. 3). These data suggested that the source of the patient’s infection was isolate B.

## Discussion

Case one highlight’s issues concerning the misdiagnosis and misreporting of a *Salmonella* Typhi isolate as a NTS by a diagnostic laboratory; with associated public health implications, as typhoid fever is a notifiable disease in most countries including South Africa. *Salmonella* Typhi has a high potential to cause outbreaks of disease [29, 30], therefore rapid diagnosis and reporting to the health authorities is vital to ensure appropriate investigation including follow up of the patient and tracing the patient’s contacts, as well as to design interventions to avert any potential outbreak.

Cases one and three highlights issues concerning the importance of accurate fluoroquinolone susceptibility testing and interpretation of results according to updated guidelines [20]. For case one, the patient was treated initially with inappropriate therapy with ciprofloxacin due to the use of defunct interpretative criteria for fluoroquinolone resistance [31], which was later followed with appropriate therapy with azithromycin. For case three, the discrepancy between the pefloxacin screening result (25 mm or susceptible) and the ciprofloxacin MIC (0.25 μg/ml or intermediately-resistant) for the isolate highlights the potential shortcomings of a single disk susceptibility screening test for fluoroquinolone susceptibility and suggests that confirmatory MIC testing should always be performed in cases of invasive *Salmonella* infections. While appropriate antimicrobial therapy does not always result in clearance of *Salmonella* Typhi from the stool, in both cases one and three, treatment with ciprofloxacin to which the isolates were intermediately-resistant failed to clear the pathogen whilst subsequent therapy with azithromycin [32] was successful.

All cases highlight issues concerning *Salmonella* Typhi vaccination of laboratory workers who are at high risk of exposure to *Salmonella* Typhi. Neither of the two laboratory workers described in cases two or three had received a *Salmonella* Typhi vaccination, despite exposures to pure cultures. Case one had received vaccine based on Vi polysaccharide antigen, but no currently available vaccine against typhoid is 100% protective; vaccination decreases risk of infection but does not completely eliminate the risk of infection. Vaccine failures are reported [33, 34]. For case one, vaccination was ineffective; vaccine failure may have occurred secondary to a faulty vaccine. In developed countries such as the USA, Canada, United Kingdom and Denmark; vaccination against *Salmonella* Typhi is offered to and encouraged for laboratory workers who are at high risk of exposure to *Salmonella* Typhi, such as those working in reference laboratories. In South Africa, this is certainly the policy and procedure at the CED at the NICD; however not all laboratory networks in South Africa provide occupation-specific vaccination. For reference laboratories in most other African countries, vaccination of laboratory workers against *Salmonella* Typhi is not routine practice. In 1980, a review of *Salmonella* Typhi laboratory-acquired infections reported that of 24 cases, the vast majority of patients (19 cases; 79.2%) had not been vaccinated; so lack of vaccination is a definite risk factor [8].

All our cases of laboratory-acquired infection were most likely the result of lapses in good laboratory practice (GLP) and laboratory safety. Case one had six years of working experience in a clinical microbiology laboratory, yet laboratory-infection still occurred. Lapses in laboratory safety do occur, even among laboratory workers with many years of experience working in a clinical microbiology laboratory. A recent report in 2015 described an NTS infection involving a laboratory technician with 20 years of experience in a clinical microbiology laboratory [18]. Over recent years, the decreasing numbers of reports concerning *Salmonella* laboratory-acquired infections probably reflect the current increased awareness and practice of GLP and laboratory safety. Risky procedures such as mouth-pipetting are now no longer practiced. Today, clinical microbiology laboratories in most countries enforce uniform safety policies nationwide, which would include: safety training; restricted laboratory access; use of biological safety cabinets; personal protective equipment (PPE) (gloves, masks, coats, etc.); hand-washing facilities with automatic-operating taps or elbow operating taps and automatic-operating paper towel dispensers; prohibiting eating, drinking and smoking in the laboratory; prohibiting cell phones in the laboratory; proper decontamination of laboratory benches before and after working with clinical specimens and bacterial cultures; etc. Laboratory accreditation to International Organization for Standardization (ISO) requirements ensures compliance and audit safety policies; diagnostic laboratories in South Africa are all accredited against the ISO 15189 standard [35]. With regards to use of biological safety cabinets, in South Africa (and most African countries), these are not mandatory for working with *Salmonella* Typhi. However, for some laboratories in Europe and the USA, it is mandatory to work with *Salmonella* Typhi within a biological safety cabinet (cabinets with a minimum of level-2 safety) or within a biosafety level-2 laboratory.

Molecular subtyping of bacterial isolates is an effective methodology to investigate the relatedness of bacterial isolates and to investigate the source of the infections. Molecular subtyping, including PFGE analysis [3, 15–18] and WGS [15], has previously been reported to investigate laboratory-acquired infections involving *Salmonella* species. In particular, Alexander and coworkers [15] used WGS to identify the source of a laboratory-acquired infection involving a laboratory technologist; interestingly, the technologist was not responsible for any testing conducted on the causative strain, so it was concluded that the infection was probably acquired while cleaning laboratory benches or discarding biohazardous waste.

For our current cases, all isolates were investigated with PFGE analysis. Although PFGE analysis is an older molecular subtyping methodology, it still provides valuable information and still remains the primary bacterial subtyping methodology employed by PulseNet International, a molecular subtyping network for foodborne and waterborne disease surveillance. PFGE analysis is limited in that it may not always provide sufficient resolution; for example, if you have a case isolate and you query a database of isolates to find a closest match by genetic relatedness, you may find that the case isolate will equally match (by PFGE pattern) a number of isolates. This was the situation for our case one, where the PFGE pattern for the case isolate was found to match a PFGE pattern represented by a cluster of isolates (Fig. 1), suggesting that the source of the laboratory worker’s infection was within this cluster of isolates that the laboratory worker had handled. To pinpoint the best possible epidemiological match, PFGE data analyzed together with epidemiological data (dates, geographic locations, patient contact information, etc.) is helpful. This was the situation for cases two and three, whereby PFGE data interpreted together with epidemiological data allowed us to pinpoint the most likely source of the laboratory worker’s infection; source isolate A for case two (Fig. 2) and source isolate B for case three (Fig. 3).

For any finer degree of resolution, alternative molecular subtyping methodologies must be employed. The ultimate methodology is WGS data analysis; this WGS methodology was used to investigate isolates for cases two and three, thereby providing a secondary analysis to the primary PFGE analysis. Our analysis of WGS data included a phylogenetic analysis based upon a comparison of SNP profiles. When the number of SNP differences is compared among isolates, the lowest number of SNP differences implies the closest genetic relationship. This allowed us to determine the exact genetic similarity between a case isolate and its probable source. For case two, the case isolate and the probable source isolate (isolate A) differed by only 41 SNPs and were located within close proximity on a phylogenetic tree (Fig. 2). For case three, the case isolate and the probable source isolate (isolate B) differed by only 6 SNPs and were located within close proximity on a phylogenetic tree (Fig. 3).

Lastly, a third molecular subtyping methodology, MLST, was employed to investigate our case isolates. MLST is generally more suitable for a long-term epidemiological comparison of isolates or a global comparison of isolates. However, in some cases of short-term epidemiological investigations (outbreak investigations), it can sometimes provide valuable supporting data. This was the situation for case three, whereby the laboratory worker had acquired his infection by exposure to two possible isolates; isolate B with MLST ST 1 or isolate C with MLST ST 2. The case three isolate presented MLST ST 1, providing evidence supporting isolate B as the cause of the infection.

## Conclusions

Laboratory-acquired infection of *Salmonella* Typhi is uncommon, as the risk of infection is low if appropriate laboratory safety policies are in place and adhered to. Nonetheless, accidental infections still do occur and would most likely be the result of lapses in aseptic techniques. In these situations, molecular subtyping of bacterial isolates is very useful to investigate the source of infections.

## Abbreviations

CDC: Centers for Disease Control and Prevention
CED: Centre for Enteric Diseases
CLSI: Clinical and Laboratory Standards Institute
CRP: C-reactive protein
DMP: Diagnostic Media Products
GLP: Good laboratory practice
ISO: Organization for Standardization
MIC: Minimum inhibitory concentration
MLST: Multilocus sequence typing
NICD: National Institute for Communicable Diseases
NTS: Nontyphoidal *Salmonella*
PFGE: Pulsed-field gel electrophoresis
PPE: Personal protective equipment
*Salmonella* Typhi: *Salmonella enterica* serotype Typhi
SNP: Single nucleotide polymorphism
ST: Sequence type
WGS: Whole-genome sequencing
